# Muscle Engagement Monitoring Using Self-Adhesive Elastic Nanocomposite Fabrics

**DOI:** 10.3390/s22186768

**Published:** 2022-09-07

**Authors:** Yun-An Lin, Yash Mhaskar, Amy Silder, Pinata H. Sessoms, John J. Fraser, Kenneth J. Loh

**Affiliations:** 1 Department of Structural Engineering, University of California San Diego, La Jolla, CA 92093, USA; 2 Active, Responsive, Multifunctional, and Ordered-materials Research (ARMOR) Laboratory, University of California San Diego, La Jolla, CA 92093, USA; 3 Department of Mechanical & Aerospace Engineering, University of California San Diego, La Jolla, CA 92093, USA; 4 Leidos, Inc., San Diego, CA 92106, USA; 5 Warfighter Performance Department, Naval Health Research Center, San Diego, CA 92106, USA

**Keywords:** electromyography, health monitoring, graphene, kinesiology tape, movement, physical performance, skin, strain sensor, wearable

## Abstract

Insight into, and measurements of, muscle contraction during movement may help improve the assessment of muscle function, quantification of athletic performance, and understanding of muscle behavior, prior to and during rehabilitation following neuromusculoskeletal injury. A self-adhesive, elastic fabric, nanocomposite, skin-strain sensor was developed and validated for human movement monitoring. We hypothesized that skin-strain measurements from these wearables would reveal different degrees of muscle engagement during functional movements. To test this hypothesis, the strain sensing properties of the elastic fabric sensors, especially their linearity, stability, repeatability, and sensitivity, were first verified using load frame tests. Human subject tests conducted in parallel with optical motion capture confirmed that they can reliably measure tensile and compressive skin-strains across the calf and tibialis anterior. Then, a pilot study was conducted to assess the correlation of skin-strain measurements with surface electromyography (sEMG) signals. Subjects did biceps curls with different weights, and the responses of the elastic fabric sensors worn over the biceps brachii and flexor carpi radialis (i.e., forearm) were well-correlated with sEMG muscle engagement measures. These nanocomposite fabric sensors were validated for monitoring muscle engagement during functional activities and did not suffer from the motion artifacts typically observed when using sEMGs in free-living community settings.

## 1. Introduction

Quantitative measurements of how muscles are engaged to enable functional movements are invaluable for diagnosing, treating, and managing musculoskeletal disorders and injuries (e.g., during physical therapy and rehabilitation). Electromyography (EMG), especially surface electromyography (sEMG), is a well-established method for assessing the health and activation of muscle fibers and muscle groups. In principle, EMG signals stem from the depolarization and repolarization of the muscle fiber cell membrane during muscle contraction. The electrophysiological action potentials generated by the transmembrane flow of positively charged sodium, potassium, and calcium ions across the concentration gradient in muscle and nerves create an electrical voltage measurable using surface or indwelling electrodes [1,2]. A single motor unit is composed of a motor neuron and the muscle fibers it innervates. Depending on the function of the muscle, a motor unit may be small (fewer fibers per neuron, indicating finer control) or large (greater fibers per neuron, indicating greater gross control). The detected voltage measured by the transducer during efferent depolarization is the summation of individual motor units [3].

sEMG is a noninvasive procedure that employs skin-mounted electrodes to detect and record muscle group electrical activity. A single electrode or an array of electrodes are placed on the skin and over the muscle(s) of interest, and the electrical activity is recorded and commonly analyzed using the root mean square (RMS) of the electrical action potential [4]. Many studies have utilized sEMG electrodes for understanding human biomechanics. For example, Elamvazhthi et al. [5] mounted sEMG electrodes on forearm muscles to establish signal signatures for various magnitudes of forearm movements. In a study by Antwi-Afari [6], spinal biomechanics during repetitive lifting tasks were measured by sEMG, and the results revealed that lifting heavier weights corresponded to increased sEMG signals in certain muscles, while other muscles did not show the same trend.

While sEMG has been used for monitoring and assessing human movements [7], several challenges still hinder its practical use. For instance, movement artifacts, which result from the relative motion between the surface electrodes and skin, between skin layers, and during stretching of skin, make these sensors difficult to use in free-living community settings [7,8,9,10]. Furthermore, muscle crosstalk during complex motion further limits the ability to assess muscle activity during complex movements, with signal quality largely dependent on sEMG sensor placement [11]. Even minimal deviations in electrode placement can induce significant signal variability [12]. For example, armband sEMG devices have poor placement repeatability during reattachment, which is inevitable in the case of long-term use [13]. On the other hand, crosstalk, which are unwanted signals picked up over non-contracted muscles or added by co-contracted muscles, have been documented as being particularly significant in gait analysis and as reducing sEMG signal fidelity [14,15,16].

Various approaches have been undertaken to improve sEMG signal quality, given the limitations of this modality. For example, Tanweer et al. [17] used inertial measurement units (IMUs) to characterize motion artifacts and correct sEMG measurements. However, similarly to many sEMG sensors, IMUs are bulky, which is inconvenient and can affect user comfort and natural movement. Mithun et al. [18] considered a wavelet-based technique for suppressing noise and EMG motion artifacts, but the amount of denoising and signal processing required are barriers to continuous, long-term, and real-time monitoring applications. Lastly, minimal crosstalk area is an experimentally-defined skin surface area where the crosstalk versus co-contraction is minimal, but these areas are difficult for non-experts to identify and thus challenging to put into practice [14,19].

The aim of this pilot study was to investigate the correlation between sEMG measurements and sensing streams from a recently developed, skin-mounted, nanocomposite, elastic fabric sensor [20]. The wearable sensor, herein referred to as “Motion Tape”, is formed by integrating piezoresistive graphene nanosheet (GNS) thin films with commercial K-Tape. The underlying principle is that greater joint exertion results in stronger contractions and corresponding changes in skin-strains. Thus, our overarching hypothesis is that Motion Tape skin-strain measurements across major muscle groups are correlated with sEMG measurements during functional movements. For the purpose of this pilot study, the aim was to establish that Motion Tape can capture the skin-strain changes associated with performing biceps curls using different weights.

## 2. Materials and Methods

### 2.1. Nanocomposite Fabric Sensor Fabrication

**Figure 1:**
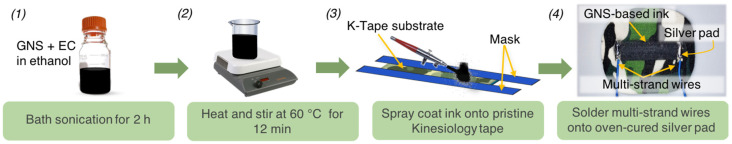
Motion Tape was fabricated by (**1**) first preparing the GNS/EC-EtOH solution and (**2**) heating while stirring it. (**3**) Spray-coating was employed to deposit GNS/EC-EtOH ink onto masked self-adhesive K-Tape substrates (**4**) to form each Motion Tape specimen.

Motion Tape was fabricated according to the procedure illustrated in Figure 1 and outlined by Lin et al. [20]. In short, Motion Tape was prepared by spray-coating GNS and ethyl cellulose (EC) thin films onto unidirectionally stretchable K-Tape (Rock Tape^®^, Durham, NC, USA). GNS was synthesized by water-assisted liquid-phase exfoliation [21]. First, a 2 *wt*.% EC solution was prepared by mixing EC in 200 proof ethyl alcohol (EtOH) and stirring the mixture for 24 h. GNS was added to the EC-EtOH solution at a concentration of 15 mg/mL and subjected to 2 h of bath sonication (150 W, 22 kHz). The dispersed GNS/EC-EtOH solution was then heated at 60 °C for ~12 min using a Thermo Fisher Scientific digital hotplate to evaporate some of the EtOH solvent while increasing viscosity. The sprayable GNS/EC-EtOH ink was obtained after it cooled to room temperature.

Second, a Paasche VL-series airbrush (Kenosha, WI, USA) was used to spray-coat the GNS/EC-EtOH ink onto K-Tape substrates that were masked, to expose only the rectangular region where the nanocomposite sensing element was desired. Spray-coating was repeated three times, while pausing ~2 min between each layer for the ink to dry after each deposition step. An additional and final layer of GNS/EC thin film was drop-casted before drying the Motion Tape specimen for at least 1 h. It was found that drop-casting enhanced the overall nanocomposite uniformity and electrical conductivity. Last, measurement electrodes were established at opposite ends of the GNS/EC sensing element by drying flexible conductive ink (Voltera, Kitchener, ON, Canada), followed by soldering of multi-strand wires (Digi-Key Electronics, Thief River Falls, Minnesota, MN, USA). The result is a self-adhesive, elastic fabric sensor that can be customized to be different sizes during fabrication and can be affixed practically anywhere on the skin.

It should be mentioned that many other studies have also proposed various skin-mounted and fabric-based sensors for assessing human movements and muscle engagement. For example, Anaya and Yuce [22] developed a portable, flexible, triboelectric-nanogenerator-based device for forearm muscles/tendons motion measurement and for assessing the motor dysfunctions associated with Parkinson’s disease. Yang et al. [23] developed a textile strain sensor by integrating carbonic ink with spandex/polyamide fabrics. The sensor successfully demonstrated highly linear, repeatable, and stable signals during various human motion monitoring events. Similarly, Reddy K et al. [24] fabricated a flexible strain sensor by coating reduced graphene oxide onto a polyester knitted elastic band. The resulting sensor showed high strain sensitivity and signal-to-noise ratios when measuring strains as small as wrist pulses and as large as knee bending movements. This work not only adds to the breadth of fabric-based skin-strain sensors already proposed but is also unique in investigating the use of a self-adhesive and unidirectionally stretchable fabric as the substrate.

### 2.2. Sensing Characterization

**Figure 2:**
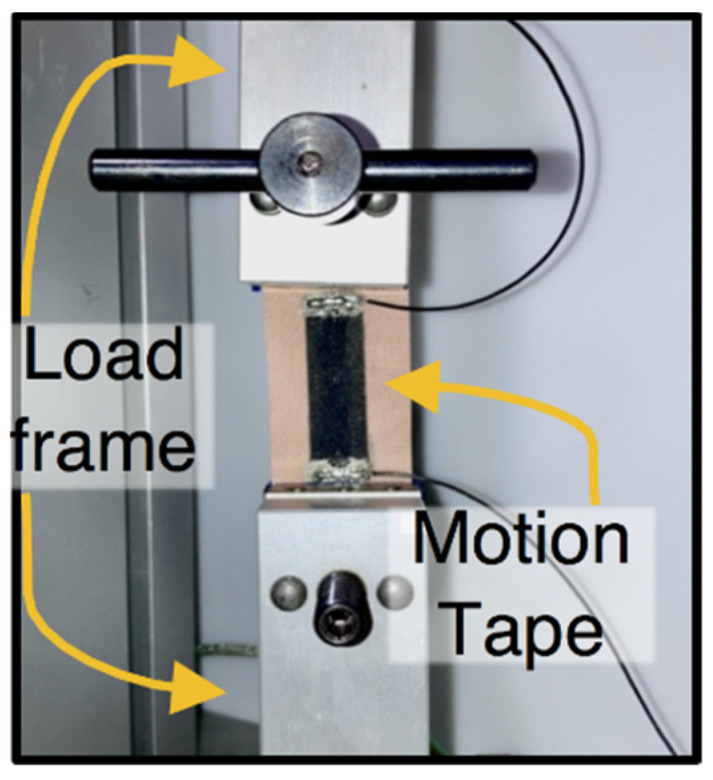
A Motion Tape specimen was mounted in a load frame for characterization tests.

Motion Tape specimens were individually mounted in a TestResources 150R (Shakopee, MN, USA) load frame for monotonic, uniaxial, and tensile cyclic electromechanical testing, as shown in Figure 2. Peak strains of 2.0%, 4.0%, 6.0%, 8.0%, and 10.0% were applied at a constant rate of 0.1 mm/s, while electrical resistance was recorded using a Keysight 34465A digital multimeter (Santa Rosa, CA, USA) sampling at 2 Hz. The load frame’s crosshead displacement and applied load were also recorded at 10 Hz. All the data were collected simultaneously using the Keysight *BenchVue* software for ease of time synchronization.

### 2.3. Human Subject Testing

**Figure 3:**
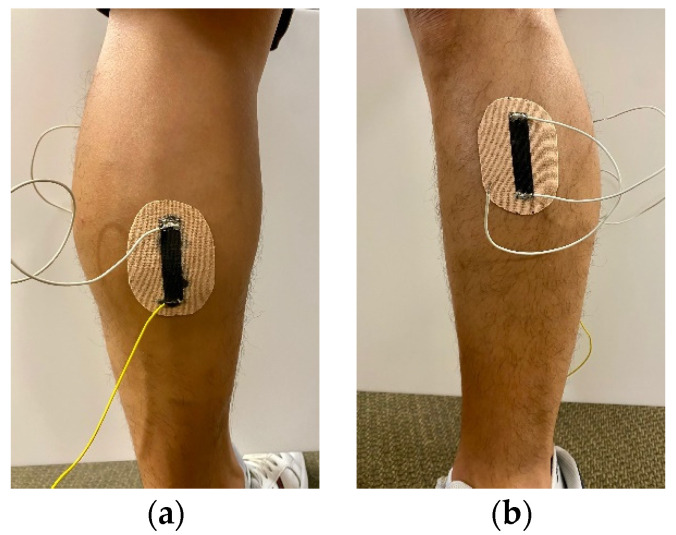
(**a**) Motion Tapes were mounted to a subject’s calf and (**b**) tibialis anterior.

Three different sets of human subject tests were conducted as part of this study. For all the tests, the adhesive backing was peeled off to directly affix, without any pre-stretching, the self-adhesive Motion Tape sensors onto skin that was pre-cleaned using an alcohol wipe. First, the sensor verification test was based on Motion Tape sensors mounted to a subject’s calf and tibialis anterior, as shown in Figure 3. Squats were performed, and the subject’s movements were captured using a 12-camera Vicon optical motion capture (mocap) system (Vicon Motion Systems Ltd., Oxford, UK), which recorded the 3D positions of all retroreflective markers at 120 Hz. The Motion Tape sensors were connected to the Vicon Lock Lab^®^ 64-channel analog interface, so that their time-synchronized electrical resistance was recorded simultaneously, at a sampling rate of 120 Hz, using the Vicon data collection software.

**Figure 4:**
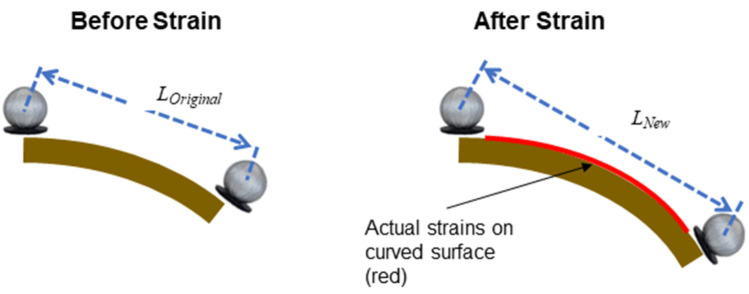
The illustration depicts errors when estimating surface strains when using the linear distance between two mocap markers.

In addition to using mocap to measure subject movements, a pair of retroreflective markers were affixed adjacent to each Motion Tape, so that the 3D positional data of the markers could be used to estimate the linear strains induced during functional movements. It should be mentioned that the calf and tibialis anterior were selected for this sensor verification test because the skin in these regions remained relatively flat during squatting, which ensured that the mocap-estimated linear strains were comparable to the skin-strains measured by Motion Tapes. However, estimating skin-strains from the change in distance between two retroreflective markers would inevitably introduce intrinsic errors. Figure 4 depicts a case when Motion Tape is mounted on a slightly curved surface (e.g., skin). When the surface is strained (e.g., due to movement or muscle engagement), the curvature of the surface would change. The actual surface strains are shown in red in Figure 4, but mocap estimates linear strains by only considering the line-of-sight change in distance between the two retroreflective markers. Strain is calculated using (*L_New_* − *L_Original_*)/*L_Original_*, which is only accurate if the strains are confined to a flat, rather than curved, surface.

**Figure 5:**
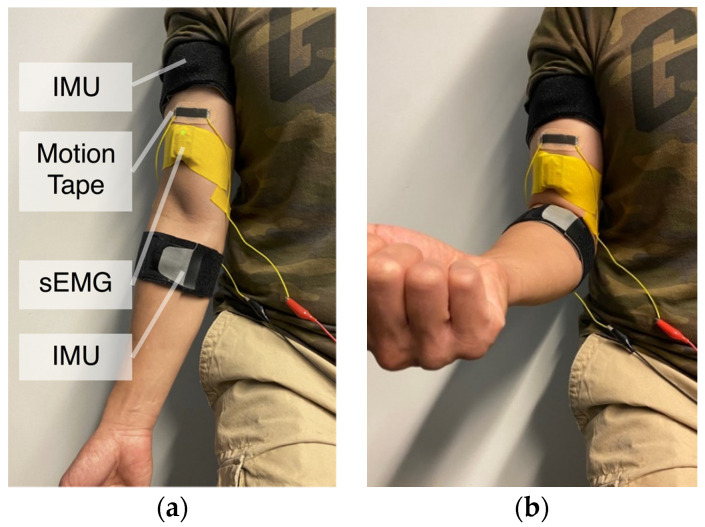
(**a**) Subjects wore Motion Tape perpendicular to the biceps brachii, along with an sEMG sensor and two IMUs, for (**b**) biceps curl muscle engagement verification tests.

The second muscle engagement human subject verification test involved participants performing biceps curls using different weights (Figure 5). Following visual cues and instruction, subjects performed biceps curls to approximately the same angle (i.e., ~51°–52°). Elbow angles during biceps curls were measured using two Xsens DOT™ IMUs (Enschede, The Netherlands) worn at the biceps and the forearm. Motion Tape was applied perpendicular to the biceps brachii, as shown in Figure 5, and its electrical resistance was recorded using a PXIe-4082 digital multimeter data acquisition (DAQ) system (National Instruments [NI], Austin, TX, USA) sampling at 296 Hz. A Delsys Trigno™ Avanti (Natick, MA, USA) wireless sEMG sensor (aluminum bar electrodes with 10-mm inter-electrode spacing) was also worn adjacent to the Motion Tape, but in parallel with the biceps brachii, to measure muscle engagement, as shown in Figure 5. The skin was also cleaned with EtOH prior to sEMG sensor attachment, which was secured on the skin using bare K-Tape. The sEMG data were recorded using the Delsys *EMGworks*^®^ analysis software running on the same personal computer (PC), to ensure time-synchronized measurements.

**Figure 6:**
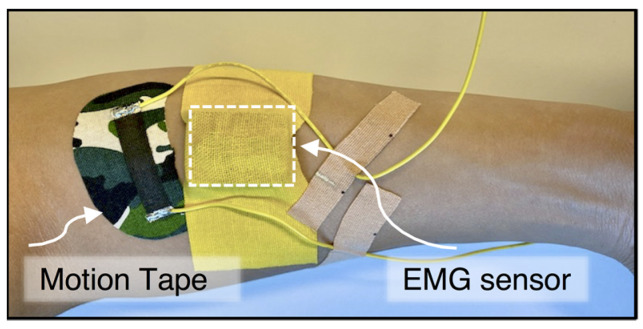
Motion Tape and an sEMG sensor were worn on a subject’s forearm while biceps curls were performed.

The third test for validating muscle engagement monitoring followed a similar protocol as the aforementioned biceps curl verification tests. Here, Motion Tape was affixed to the forearm, perpendicular to the flexor carpi radialis, as shown in Figure 6, and its electrical resistance was also recorded using the NI DAQ system. A Delsys Trigno Avanti sEMG sensor was attached adjacent to the Motion Tape but in parallel with the flexor carpi radialis (Figure 6). Biceps curls were performed using different weights, while the sensing streams from both the Motion Tape and sEMG were recorded simultaneously.

## 3. Results and Discussion

### 3.1. Strain Sensing Properties

The hypothesis that Motion Tape could capture skin-strain measurements correlated with the degree of muscle engagement was tested in this study. First, the strain sensing properties of Motion Tape were verified by subjecting them to tensile cyclic loads to different peak strains while simultaneously measuring their electrical resistance (Figure 2). Here, a 10% strain limit was selected, because the expected skin-strains over major muscle groups should be below this threshold, unlike joints, which can be greater than 30%.

**Figure 7:**
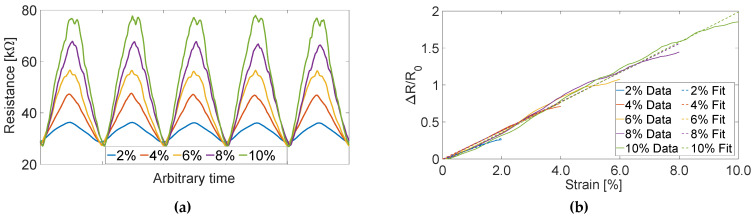
(**a**) Motion Tape resistance time history measurements with different peak strains (from 2% to 10%) show its stable and repeatable sensing response. (**b**) Linearity was confirmed by fitting linear least-squares regression lines (dashed lines) to the normalized change in resistance data (solid lines) with respect to the applied strains.

The electrical responses of tests loaded to different peak strains are overlaid in Figure 7a. Since all the tensile cyclic tests were conducted at the same loading rate of 0.1 mm/s, the overlaid plots in Figure 7a were produced by normalizing the time scale (*x*-axis) with respect to the shortest duration test (i.e., 2% peak strain). This post-processing step allows one to directly compare Motion Tape sensing response corresponding to different peak strains. Overall, the set of representative resistance time histories acquired during tensile cyclic testing confirmed the Motion Tape’s stable and repeatable strain sensing behavior (Figure 7a). In addition, sensor linearity was assessed by normalizing its change in resistance (∆*R*) with respect to its unstrained baseline resistance (*R*_0_) and then plotting the data against applied strains, as shown in Figure 7b. Linear least-squares regression lines fitted to the data confirmed sensor linearity (with correlation coefficients, *ρ*, that all exceeded 0.99) and consistent sensitivity, which ranged from 13.0 to 18.5. These results are consistent with the findings presented by Lin et al. [20], where Motion Tape sensor linearity and sensitivity remained constant, with no baseline resistance drifts, even after >200 cycles of cyclic loading.

### 3.2. Verification of Motion Tape for Skin-Strain Monitoring

Verification tests of skin-strain monitoring during functional movements were performed with two participants wearing Motion Tapes at the calf and tibialis anterior, which was described in Section 2.3. The sensor verification tests were performed by affixing a pair of retroreflective markers adjacent to each Motion Tape placed on the calf and tibialis anterior. An optical motion capture system recorded their 3D positions during squatting motion. The linear strain of each Motion Tape was estimated from changes in marker pair distances with respect to their initial separation distance when the subject stood still (Figure 4) [25]. Muscle engagement verification tests were conducted by placing an sEMG electrode over the upper arm and forearm so that the Motion Tape electrical resistance could be compared against muscle engagement during biceps curls.

**Figure 8:**
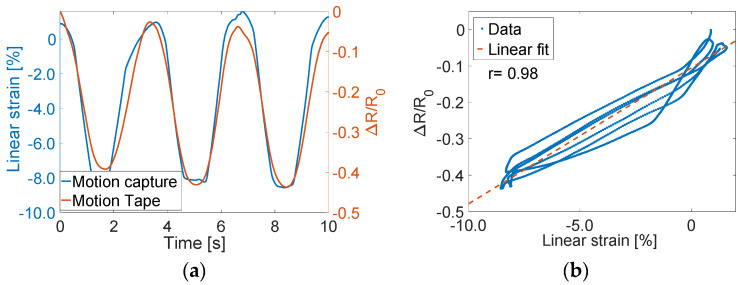
(**a**) The normalized change in resistance time history of Motion Tape on the calf is overlaid with linear strains estimated from optical motion capture. (**b**) Linear and low-hysteresis compressive strain sensing was confirmed.

Motion Tape electrical resistance and mocap data were first collected when subjects performed squats. Figure 8a plots the normalized change in resistance time history of the Motion Tape on the calf (Figure 3a), and this dataset was also overlaid with the mocap-estimated linear strains. Mocap confirmed that compressive skin-strains were induced due to contraction of the gastrocnemius medialis (i.e., calf muscle), which corresponded to decreasing the normalized change in resistance (*R_n_* = ∆*R*/*R*_0_) of Motion Tape as the subject moved from a standing to a squatting posture. Plotting *R_n_* with respect to linear strains verified its linear and low-hysteresis sensing performance, which was supported by the high correlation coefficient (*ρ* = 0.98) of the fitted linear least-squares regression line in Figure 8b. No apparent baseline resistance drifts were observed.

**Figure 9:**
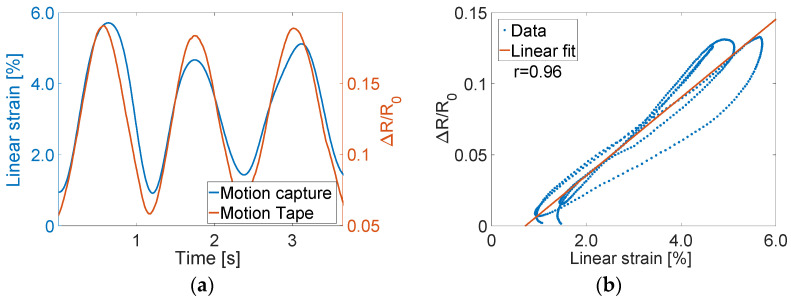
(**a**) The time history results for Motion Tape on the tibialis anterior are overlaid (**b**) with similar strain sensing properties observed with a tension similar to the compression case.

The same conclusions could be drawn from the data collected from mocap and Motion Tape mounted on the tibialis anterior during squatting (Figure 3b). Tension was induced in the tibialis anterior, which caused *R_n_* to increase (as opposed to decreasing on the calf), as shown in Figure 9a. Strong linear skin-strain sensing response was also observed, where *ρ* = 0.96 based on the best-fit linear least-squares regression line shown in Figure 9b. However, in both cases, Figure 8b and Figure 9b show some hysteresis response. This hysteresis may be an artifact from errors associated with estimating linear strains from mocap, which was explained in Section 2.3 and Figure 4. While the Motion Tape characterization tests did not reveal any hysteresis behavior (Figure 7), the sensors could potentially exhibit hysteresis at high strain rates (such as the case during these movements). Future tests will investigate if and how Motion Tape behavior changes during different applied strain rates during intense physical activities.

**Figure 10:**
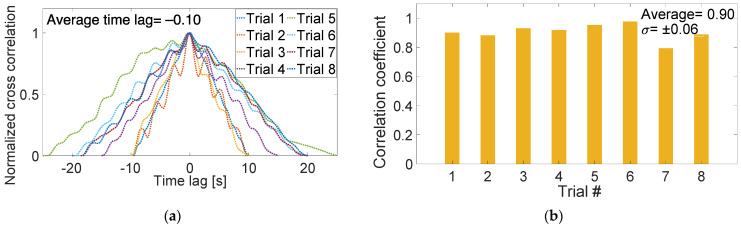
(**a**) The normalized cross-correlation between Motion Tape and motion capture estimated strains confirmed a low time lag. (**b**) The subject tests were repeated in eight trials, and all the results suggested a strong linear skin-strain sensing response.

In fact, further analyses were also performed to examine the Motion Tape’s sensing response among eight different trials. Normalized cross-correlation was applied between the Motion Tape *R_n_* and mocap-estimated linear strain time histories. The results in Figure 10a show that the average time lag was 0.10 s, suggesting nearly instantaneous Motion Tape response to changes in skin-strains. The correlation coefficients for all eight trials are also summarized in Figure 10b. The average *ρ* was 0.90, with a standard deviation of ±0.06, again confirming a strong sensor linearity and Motion Tape’s ability to accurately quantify skin-strains.

### 3.3. Verification of Motion Tape for Measuring Muscle Engagement

Building upon the skin-strain sensing verification test results presented in Figure 8 and Figure 9, biceps curl subject tests were performed to test the hypothesis that Motion Tape could measure muscle engagement during functional movements (see Section 2.3). In addition to mounting Motion Tape over the biceps brachii, a wireless sEMG sensor was also worn in parallel with the biceps brachii to measure muscle activation (Figure 5). Two IMUs were also attached to the forearm and upper arm to measure the change in elbow angle during biceps curls. Each subject then performed biceps curls using three differently weighted dumbbells, while maintaining approximately the same range of movement (as confirmed by IMU-derived angles of rotations). Although sEMGs and IMUs can both suffer from movement artifacts, especially during high-intensity activities, the biceps curls were performed slowly to avoid such experimental errors.

**Figure 11:**
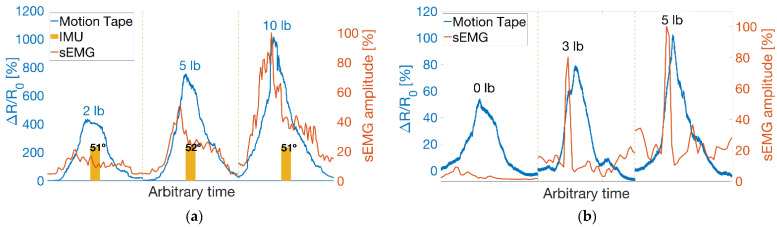
(**a**) Motion Tape Rn time histories are overlaid with sEMG measurements when a subject lifted 2, 5, and 10 lb dumbbells. (**b**) The biceps curl results from a different subject and test using 0, 3, and 5 lb weights are also shown.

Figure 11a overlays Motion Tape and sEMG data for biceps curls performed using 2, 5, and 10 lb dumbbells, where the maximum angle of rotation was maintained at 51°–52°. The sEMG data shown were denoised using an RMS calculation based on a moving window (0.125 s window length and 0.0625 s window overlap) [26], and the data were also normalized with respect to the peak voltage amplitude (i.e., results are displayed from 0% to 100%). Figure 11a shows that Motion Tape measured larger skin-strains on the biceps as heavier weights were lifted. The same trend and greater muscle engagement were also recorded by sEMG.

To show that these trends are not unique for a particular subject, Figure 11b plots similar results when a different subject performed biceps curls using 0, 3, and 5 lb weights. Similar trends were observed, with both the Motion Tape skin-strains and sEMG signal peaks increasing when heavier weights were lifted. The 0 lb case also showed changes in skin-strains (i.e., much greater than the sEMG signal changes), but this is expected because Motion Tape is a skin surface measurement and does not solely measure muscle engagement. The correlation between Motion Tape skin-strain measurements and sEMG signals suggest that Motion Tape is sensitive enough to capture skin-strain features associated with different degrees of muscle engagement. Furthermore, a comparison of Figure 11a,b shows that the magnitude of *R_n_* varies between different subjects. This is expected, given that muscle properties, body fat, and other subject-specific parameters would influence Motion Tape outputs, as they would for sEMG signals as well.

### 3.4. Validation of Motion Tape for Measuring Muscle Engagement

**Figure 12:**
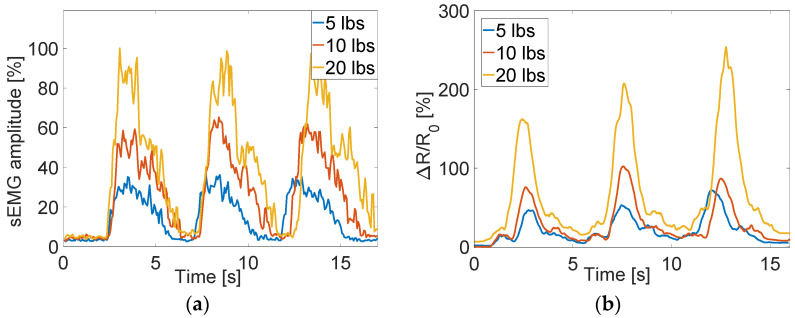
(**a**) The sEMG results showed greater muscle activation when heavier weights were lifted, and (**b**) the corresponding Motion Tape results exhibited the same trends.

Additional Motion Tape and sEMG tests were conducted on a different muscle group to show that the sensing principle applies elsewhere on the body (Section 2.3). Both types of sensors (i.e., Motion Tape and sEMG) were mounted on a subject’s forearm (flexor carpi radialis), as shown in Figure 6. Biceps curls using 5, 10, and 20 lb dumbbells were performed while maintaining a similar range of movement. The sEMG signals of Figure 12 confirm a greater muscle activation as heavier weights were lifted, and the Motion Tape *R_n_* time histories also showed greater amplitudes, following a similar trend. These results successfully validated that Motion Tape skin-strain measurements are well-correlated with sEMG muscle engagement measurements. However, it should be noted that the biceps brachii and flexor carpi radialis are major muscle groups. Other and smaller muscle groups will be considered in future tests, to further validate this technology, as well as during other types of functional movements.

## 4. Conclusions

In summary, a self-adhesive, low-profile, conformable, and disposable wearable skin-strain sensor for muscle engagement monitoring was presented. Experiments on Motion Tape conducted using a load frame and on human subjects successfully verified their linear, stable, and repeatable strain sensing properties. To show that Motion Tape could measure different degrees of muscle engagement during functional movements, biceps curl tests were performed with subjects also wearing an sEMG sensor. Sensor data from directly mounting Motion Tape over the biceps brachii, as well as over the flexor carpi radialis (i.e., forearm), showed that greater muscle activation (i.e., higher amplitude sEMG signals) was correlated with greater changes in Motion Tape resistance. It is worth noting that, while sEMG is considered the gold standard for muscle engagement monitoring, it is relatively rigid and susceptible to motion artifacts when not secured properly to the skin, whereas Motion Tape can remain affixed firmly in place on the skin while deforming freely. Overall, these wearable textile-based sensors and their continued testing and development could lead to their potential use for sports coaching, physical rehabilitation, and telemedicine, among many other healthcare, athletic, and military applications. While Motion Tape cannot replace sEMGs for quantifying muscle activation, these sensing streams can potentially provide insights about the degree of muscle engagement, especially during movement-intensive activities. Future work will focus on clinical studies that rigorously test Motion Tape for muscle engagement monitoring in different body areas, among diverse subjects, and with larger subject pools.

## 5. Patents

K.J. Loh and Y-A. Lin, “Smart Elastic Fabric Tape for Distributed Skin Strain, Movement, and Muscle Engagement Monitoring,” U.S. Utility Patent Application 17/379,522. Submitted on 19 July 2021.

## Data Availability

Data are available upon written request submitted to the corresponding author.

## Disclaimer

A.S., P.H.S. and J.J.F. are employees of the U.S. Government. This work was prepared as part of their official duties. Title 17, U.S.C. §105 provides that copyright protection under this title is not available for any work of the U.S. Government. Title 17, U.S.C. §101 defines a U.S. Government work as work prepared by a military service member or employee of the U.S. Government as part of that person’s official duties. Report No. 22-27 was supported by the Office of Naval Research (ONR) under grant no. N00014-20-1-2329 (principal investigator: Kenneth J. Loh). The views expressed in this article are those of the authors and do not necessarily reflect the official policy or position of the University of California San Diego, the Department of the Navy, Department of Defense, nor the U.S. Government. The study protocol was approved by the University of California San Diego, in compliance with all applicable Federal regulations governing the protection of human subjects. Research data were derived from a University of California San Diego Institutional Review Board protocol, number 191806.
